# Visceral leishmaniasis in selected communities of Hamar and Banna-Tsamai districts in Lower Omo Valley, South West Ethiopia: Sero-epidemological and Leishmanin Skin Test Surveys

**DOI:** 10.1371/journal.pone.0197430

**Published:** 2018-05-24

**Authors:** Fitsum Bekele, Tariku Belay, Ahmed Zeynudin, Asrat Hailu

**Affiliations:** 1 Department of Medical Laboratory Sciences, College of Medicine and Health Sciences, Wolkite University, Wolkite, Ethiopia; 2 Department of Medical Laboratory Sciences and Pathology, College of Public Health and Medical Sciences, Jimma University, Jimma, Ethiopia; 3 Department of Microbiology, Immunology & Parasitology, Faculty of Medicine, Addis Ababa University, Addis Ababa, Ethiopia; Universidade Federal da Bahia, BRAZIL

## Abstract

**Background:**

Visceral leishmaniasis [VL] is a debilitating parasitic disease which invariably kills untreated patients. The disease is caused by *Leishmania (L*.*) donovani or L*. *infantum*, and transmitted by the bite of female phlebotomine sandflies. VL often remains subclinical but can become symptomatic with an acute/subacute or chronic course. Globally, the Eastern Africa region is one of the main VL endemic areas. The disease is prevalent in numerous foci within Eritrea, Ethiopia, Kenya, Somalia, Sudan South Sudan, and Uganda. In Ethiopia, the Lower Omo plain is one of the many VL endemic regions.

**Objectives:**

The objective of this study was to determine the prevalence of asymptomatic visceral leishmaniasisin Hamar and Banna-Tsamai districts of the South Omo plains where VL is becoming an emerging health problem of neglected communities.

**Methods:**

A community based cross-sectional survey was conducted in 2013 between 25th of July and 14^th^ of August. A total of 1682 individuals living in 404 households were included in the study. Socio-demographic and clinical data were collected from each of the participants and venous blood was also collected for the detection of antibodies to visceral leishmaniasis using Direct Agglutination Test. Leishmanin Skin Test was performed to detect the exposure to the parasite.

**Results:**

The surveys included 14 villages located in areas where VL had been reported. In a study population of 1682 individuals, the overall positive leishmanian skin test and sero-prevalence rates respectively were 8.6% and 1.8%. A statistically significant variation in the rate of positive LST response was observed in different study sites and age groups. Positive LST response showed an increasing trend with age. The sero-prevalence rate also showed a statistically significant variation among different study sites. Higher rates of sero-prevalence were observed in children and adolescents. The LST and sero-prevalence rates in Hamar District exceeded significantly that of Banna-Tsamai District (10.7% versus 5.8% for LST; and 2.6% versus 0.7% for sero-prevalence).

**Conclusion:**

The prevalence of asymptomatic VL infection in Hamar and Banna-Tsamai districts during the study period in 2013 was low compared to rates previously reported in other endemic areas of Ethiopia. This could be due to the fact that the disease is emerging in Hamar and Banna-Tsamai districts. Based on records of a nearby Hospital, increasing numbers of active VL cases have been reported in these districts through the years 2006–2012, especially in Hamar District. Both districts are important destinations of tourism, and thus the importance of surveillance should be emphasized. Detailed epidemiological and entomological studies are recommended.

## Background

VL is a parasitic disease caused by species of the *Leishmania donovani* complex [1]. Following a successful bite and inoculation by the female phlebotomine sandfly, the parasite enters macrophages and establishes the infection [2].

The WHO estimates that worldwide about 500,000 new cases of VL occur every year [1]; 90% of which is borne by 6 countries: India, Bangladesh, Sudan, South Sudan, Brazil and Ethiopia [3]. In global estimates; Sudan, South Sudan, Ethiopia, Kenya, and Somalia accounts for the second largest number of annual VL cases, after South Asia [4, 5]. Within the Eastern Africa region, VL is prevalent in many foci in Eritrea, Ethiopia, Kenya, Somalia, Sudan, South Sudan and Uganda where the number of VL cases has increased markedly in the past decade [4, 6].

In the horn of Africa, Ethiopia is the third most affected country with an annual incidence of 3700–7400 cases [3]. The disease is known to be endemic in Metema and Humera plains in north west; in several localities of south western Ethiopia, i.e., the Omo plains, the Aba Roba focus in Segen valley, and Woito River valley adjacent to South Omo; in southern Ethiopia around Moyale area close to the borders with North Kenya; and in south eastern Ethiopia around Genale river basin in Oromia Regional State, Afder and Liban zones in Somali Regional State [4, 6].

**Fig 1:**
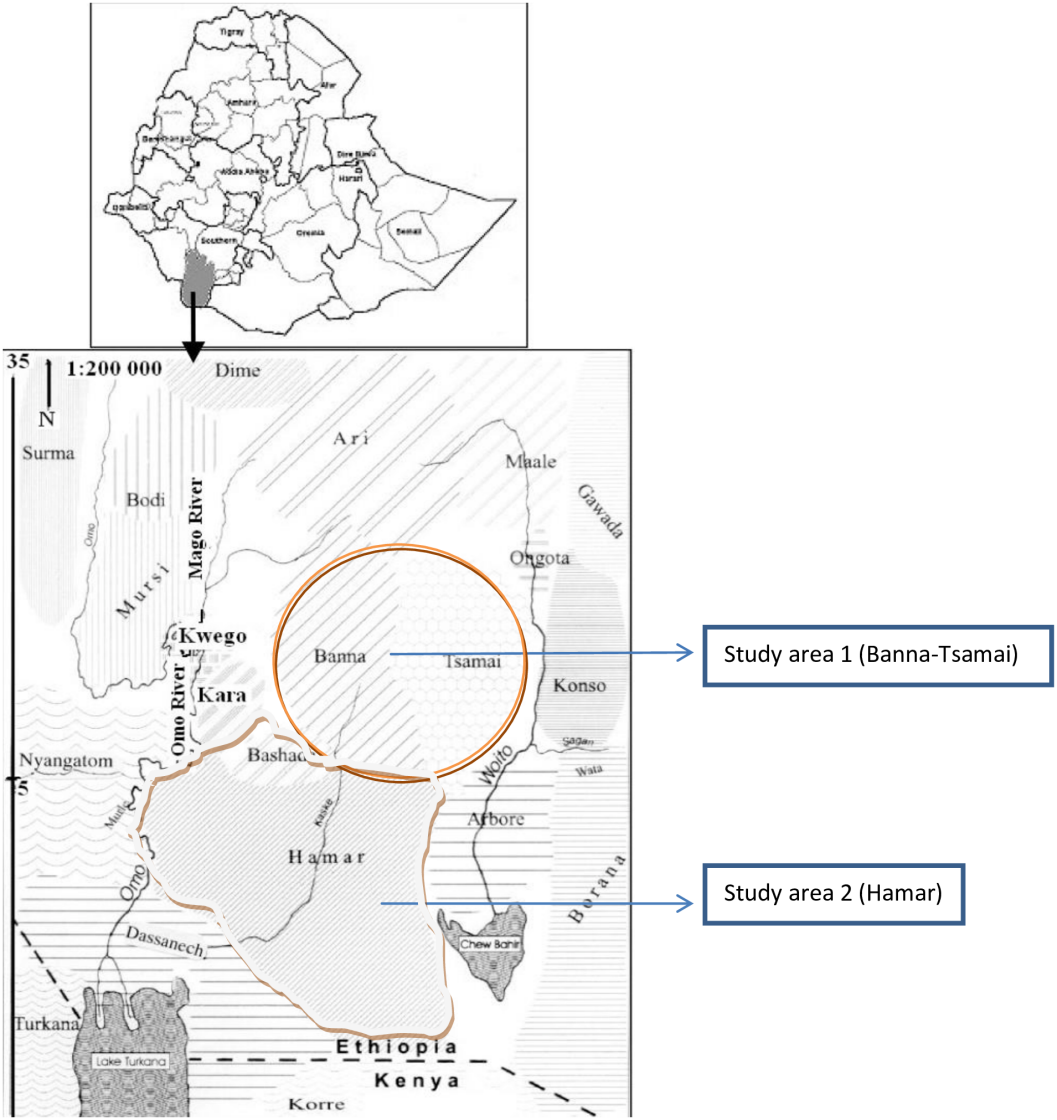
Map of Lower Omo basin in south western Ethiopia southing study districts (Hamar & Banna-Tsamai) as well as neighboring districts (Nyangatom, Dassanech, Konso, Ari and Arbore).

Hamar and Banna-Tsamai districts, localities in Lower Omo Plains (Fig 1), adjoin neighboring VL endemic localities in south west Ethiopia; i.e., the Segen and Woito river valleys. These districts are also close to the areas where an outbreak of VL was reported in a battalion of British troops [7].

The people living in Hamar and Banna-Tsamai districts are among those minority communities who do not have access to VL diagnosis and treatment services. Historically, the Lower Omo Plains has been a sparsely populated region, where VL used to occur only sporadically in areas west of Hamar and Banna-Tsamai districts affecting mainly children in the Nyangatom and Dasanetch communities. Recent observations show that VL in Hamar and Banna-Tsamai districts is emerging as a threat to community health, tourism and rural agro-industrial development. An ongoing sugarcane agro-industrial development scheme around Omo River that has brought significant demographic changes in the region is expected to impact the epidemiology of VL. Such has happened in early 70’s and mid 90’s in northwest Ethiopia where VL is presently a major public health problem.

Due to the increasing number of VL cases reported in Hamar District, we launched an epidemiological study of VL in Hamar and Banna-Tsamai districts, beginning with a study on prevalence of asymptomatic infections.

## Methods

### Study design

A community-based cross-sectional survey was conducted in the period 25 July– 14 August 2013. The leishmanin skin test (LST) and direct agglutination test (DAT) were employed to measure exposure to leishmania and to determine prevalence of asymptomatic infection.

### Study area

The study was conducted in Hamar and Banna-Tsamai districts, Lower Omo Valley, south west Ethiopia. Hamar Woreda (District) covers an area of 5,697.38 sq. km and had 24 kebeles (lowest administrative units in Ethiopia), of which 13 had previously reported VL. All the study kebeles in Hamar are found below 1000m above sea level, the lowest being Cherqeqa at 550m. Based on demographic figures published by the Central Statistical Agency (CSA) of Ethiopia in 2007, this Woreda had an estimated total population of 56,359.

Banna-Tsamai is a district adjacent to Hamar, covering an area of 2,922.76 sq. km and had 23 kebeles during the study, among which 3 kebeles (namely Olu, Luka and Birayle) had reported VL in the past. Olu is found 1285m above sea level whereas Luka is at 630m. According to CSA 2007 report, the district had an estimated total population of 50,814.

Of the 24 kebeles in Hamar and 23 in Banna-Tsamai, 16 (13 in Hamar and 3 in Banna-Tsamai) had reported 48 cases of VL in the years 2006–2012 (unpublished reports; Tables A & B in S1 Table) The 13 kebeles in Hamar reported 43 VL cases among individuals living in 19 villages (clusters). Similarly the 3 kebeles in Banna-Tsamai had reported 5 cases of VL among individuals living in 4 villages.

### Sample size

Sample size was calculated by using a single population proportion formula taking 50% as estimate of prevalence and a 3% margin of error. With a design effect of 1.5 and considering a10% non-response rate, the final sample size was calculated to be 1760.

### Sampling sites and sampling technique

The sampling sites were selected in kebeles where VL cases were recently reported. Kebeles were identified by reviewing case records of Arbaminch Hospital Leishmaniasis Research and Treatment center [AMH-LRTC]. Based on the 2006–2012 patient records, we identified 13 kebeles of Hamar and 3 kebeles of Banna-Tsamai as potential sampling sites. Further selection of specific sampling sites at a village levelwas based on the criteria that: 1) a kebele should have villages that reported a minimum of 2 VL cases in the years 2006–2012; and 2) a kebele should comprise villages that are located in rural settlements. These criteria excluded 9 kebeles whosevillages had each reported only a single case of VL between 2006 and 2012. Two other kebeles (Turmi and DimekaZuria, Tables A & B in S1 Table) were urban settings, and thus were also excluded. Accordingly; 3 kebeles (Besheda, Cherqeqa, Sinbile) in Hamar and 2 kebeles in Banna-Tsamai (Olu and Luka) were the selected study kebeles (S2 Table). In these 5 kebeles, 14 villages/clusters were identified to comprise sampling sites based on a sample size of 1760 individuals. To include a study population of 1760 individuals, approximately 440 households were needed (average household size = 4). The 440 households were distributed across the 14 villages, proportional to the number of households in each village. Households were then randomly selected using the register that was available in the kebele office.

### Questionnaire and clinical examination

Socio-demographic characteristics were collected using pre-structured questionnaires. An enrollment form containing past medical history, medical complaints and other demographic data was completed for every individual. A general clinical examination was performed in each individual with particular reference to hepatosplenomegaly, presence/absence of fever or recent history of fever, enlargement of lymph nodes and presence of scars of previous cutaneous leishmaniasis and/ or post kala azar dermal leishmaniasis [PKDL]. In sick looking subjects having complaints of abdominal pain/swelling and/or fever and weight loss, liver size was measured in the mid-clavicular line from the costal margin; and the spleen size was assessed by measuring the distance between the coastal margins in the anterior axillary line to the tip of the spleen. Lymphadenopathy was classified as localized if found only at one site and generalized if found in two or more sites [5].

### Sample collection and processing

The total blood volume collected was 5 ml from adults and 3 ml from children. The rk-39 rapid diagnostic test was performed while in the field, whereas Direct Agglutination Test was performedin the facilities of Leishmaniasis Research and Diagnostic Laboratory-Addis Ababa University (LRDL-AAU).

### Leishmanin Skin Test

**Fig 2:**
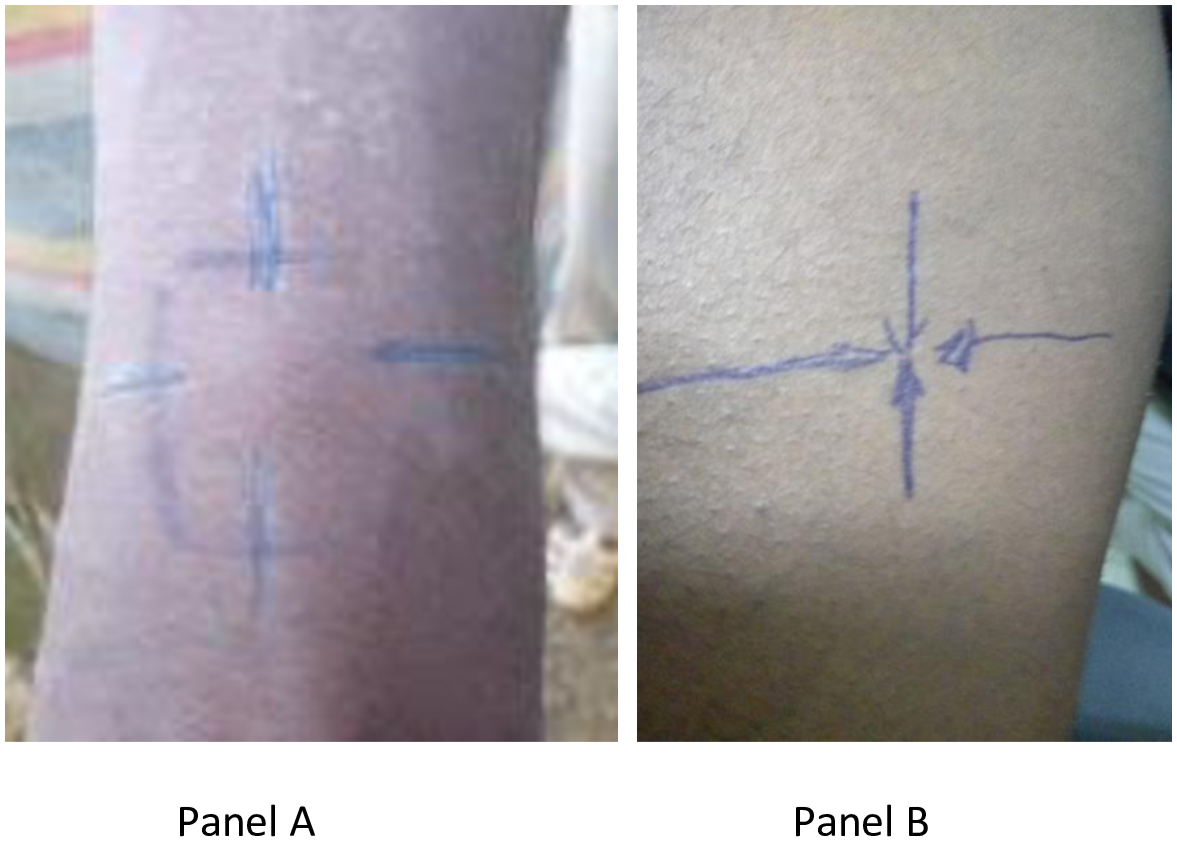
Postive (Panel A) and negative (Panel B) LST reactions.

The Leishmanin skin test (LST) is a test for Delayed Type Hypersensitivity (DTH) reaction specific to leishmania parasites, also known by the name Montenegro Test. The antigen (lot # 126-3/06-2010) prepared from *L*. *major* MRHO/IR/75/ER was kindly provided by Dr. Alimohammadian, Pasteur Institute of Iran. In this test, 0.1 ml of a 5 × 10^6^/ml suspension of leishmania promastigotes prepared in pyrogen-free phosphate buffered saline was injected on the volar aspect of the forearm. After 48 to 72 hours, the size of indurations was measured using the ball point method as previously described [8]. Results were recorded as average of two dimensional readings (Fig 2). Induration size of 5.0 mm and above were considered positive [8]. This L. major based leishmanin antigen had previously been used in South and Eastern Ethiopia in VL surveys [8–11].

### Direct Agglutination Test (DAT)

**Fig 3:**
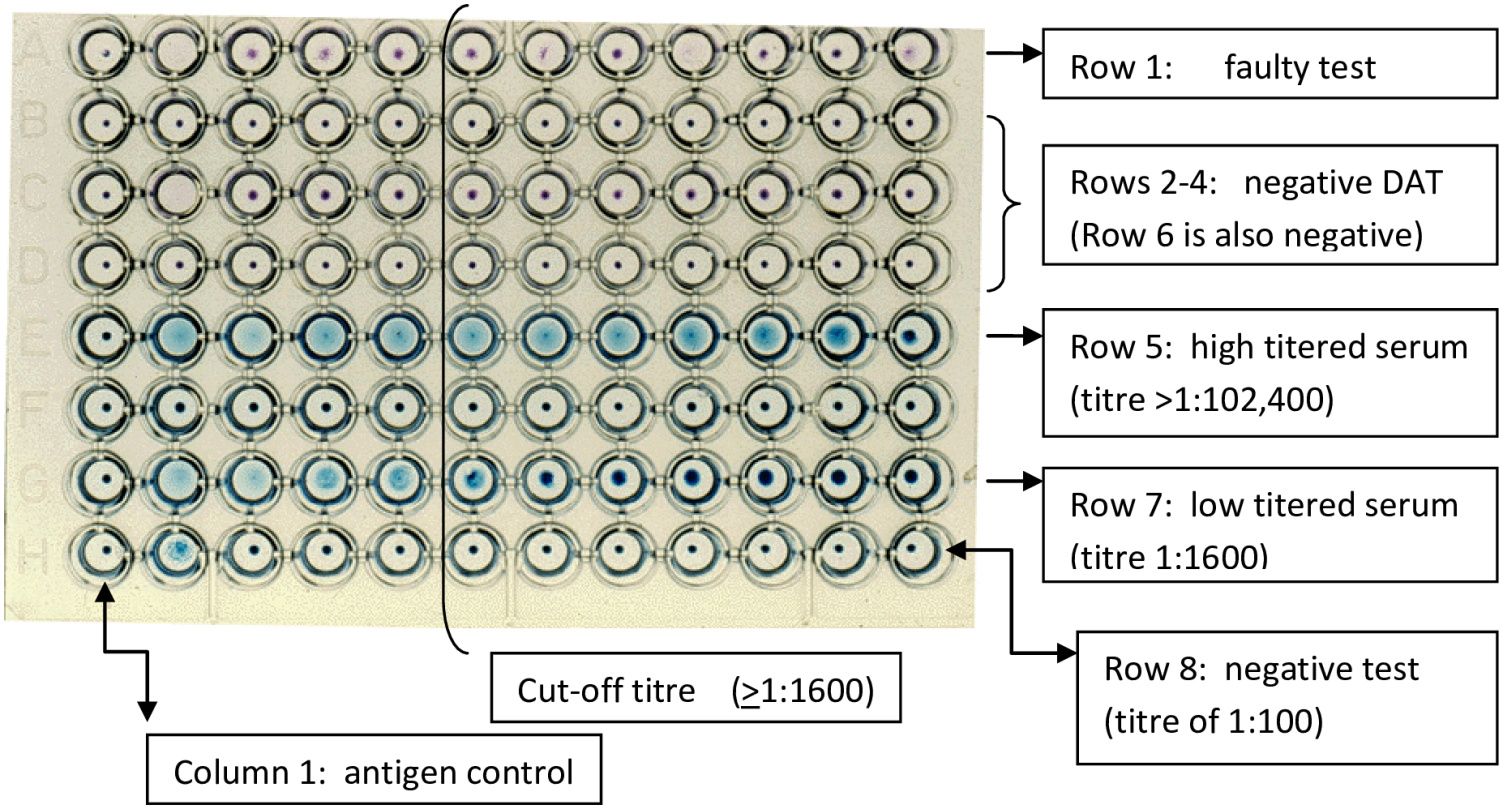
A plate showing DAT test results in sera tested with a starting dilution of 1:100 in column 2.

Blood samples were collected by venipuncture from a peripheral vein. Serum was separated by centrifugation from coagulated blood. DAT was performed as previously described using a freeze-dried antigen obtained from KIT [12]. In brief, serially diluted serum samples were incubated in v-shaped micrtotitre plates with killed coomassie blue stained and fixed promastigotes of *Leishmania donovani* prepared from MHOM/SD/68/IS [12]. The plates were incubated at room temperature for 8–12 hours and then read visually. In serum samples that did not contain anti-*Leishmania* antibodies, the antigen sediments to the bottom of the well and formssharp blue dots; and in those where anti-*Leishmania* antibodies were present, agglutination was visible as a mat, or a dot with frayed edges or an enlarged dot [5, 12] as depicted in Fig 3. DAT is a semi-quantitative test requiring a cut-off titre for determining positive and negative test results. A cut-off titre 1:1600 (Fig 3) was used in this study as previously recommended for Ethiopia [13].

### Rk-39 immunochromatographic test

This is a simple field adapted immunochromatographic test used for rapid diagnosis of VL. We used DiaMedITLeish (supplied by DiaMed AG, BioRad, USA) for testing clinically suspected cases in the field, where results become available within 20 minutes. The test was not intended for use in detection of asymptomatic infections.

### Data analysis

Data was entered into an excel sheet, checked for completeness, exported to SPSS version 20 and Epi Info 7, and analyzed by the same. Chi-square and the corresponding p-values were used to determine the statistical significance of the proportions/ratios obtained from the cross tabulated data. A p-value < 0.05 was considered statistically significant. Odds Ratio and the 95% CI were computed from a 2x2 table using Epi Info 7.

### Ethical consideration

Ethical clearance was obtained from Jimma University Research and Ethics Review Committee. Permission was also obtained from the respective Woreda (District) Health Bureaus in Hamar and Banna-Tsamai. A written informed consent was sought from each individual prior to involvement in the study. For children, their parents were asked to involve them in the study, and to give consent on their behalf. Patients who fulfilled case definition of VL and became positive on rk-39 immunochromatographic test were sent to Arbaminch Hospital for detailed physical and laboratory examinations, and treatment for VL upon confirmation of the diagnosis.

## Results

### Characteristic of the study population

A total of 1682 (S2 Table); i.e., 96.6% of the planned 1760 individualsliving in 404 households; i.e., 925(55.0%) females and 757(45.0%) males from different age groups, were included in the study. The minimum age of an individual included in the study was 18 months. Each study subject had undergone physical examination, LST and DAT. LST was administered to1513 subjects, of which 1220 had returned after 48–72 hours for reading induration sizes of the DTH reactions to leishmanin; whereas all 1682 individuals had been tested for the presence of antibodies to leishmania using DAT. The majority of the study subjects (58%) were from Hamar Woreda and the remaining (42%) were from Banna-Tsamai. Majority of the study subjects (40.7%) were less than 10 years of age followed by 10–19 years of age (23.0%). Those aged 60 and above were 2.5% of the study subjects.

### Active cases of VL

Among the 1682 individuals examined, there were 23 individuals who had palpable spleen or hepatosplenomegaly. The 23 individuals claimed a recent history of fever, while three had an observed fever. All the 23 individuals were tested by rk-39 immunochromatographic test, of which two were positive and sent to Arbaminch Hospital for further confirmation of diagnosis. The patients were treated with sodiumstibogluconate at 20mg/kg body weight for 30 days following parasitologic confirmation.

### Leishmanin Skin Test

LST and DAT positivity ratesshowed variation by geographic location (Table 1), age groups (Table 2) and gender (Table 3). The overall LST positive rate was 8.6% (8.2% in males, 9.0% in females).

**Table 1:** Prevalence of leishmania infection/exposure determined by positive LST and DAT rates in different kebeles and villages, Hamar and Banna-Tsamai districts of South Omo region, South West Ethiopia, July 2013.

Kebeles/villages	LST		DAT	
	# tested	# (%) positive	# tested	# (%) positive
**Besheda**	**261**	**16(6.1)**	**386**	**8(2.1)**
• Argude	195	15(7.7)	258	8(3.1)
• Gune	66	1(1.5)	128	0(0.0)
**Cherqeqa**	**282**	**54(19.1)**	**376**	**17(4.5)**
• Ayro	127	19 (15.0)	169	8(4.7)
• Wisina	155	35(22.6)	207	9(4.3)
**Sinbile**	**147**	**4(2.7)**	**209**	**0(0.0)**
• Ago	64	3(4.7)	98	0(0.0)
• Bashaw	58	0(0.0)	77	0(0.0)
• Bere	25	1(4.0)	34	0(0.0)
**Luka**	**347**	**22(6.3)**	**484**	**4(0.8)**
• Babo	123	5(4.1)	156	2(1.3)
• Luka	141	17(12.1)	213	0(0.0)
• Seliya	83	0(0.0)	115	2(1.7)
**Olu**	**183**	**9(4.9)**	**227**	**1(0.4)**
• Bahe	52	4(7.7)	66	0(0.0)
• Guge	22	0(0.0)	25	0(0.0)
• Sala	55	2(3.6)	70	1(1.4)
• Shirgo	54	3(5.6	66	0(0.0)
**Total**	**1220**	**105 (8.6)**	**1682**	**30 (1.8)**
Statistic	X^2^ = 74.5;p<0.001		X^2^ = 31.8;p<0.001	

**Table 2:** Prevalence of leishmania infection/exposure determined by positive LST and DAT rates in different age groups, Hamar and Banna-Tsamai districts of South Omo region, South West Ethiopia, July 2013.

Age group	LST		DAT	
	# tested	# (%)positive	# tested	# (%)positive
<10	511	4(0.8)	684	12(1.8)
10–19	274	17(6.2)	386	10(2.6)
20–29	143	31(21.7)	203	5(2.5)
30–39	124	26(21.0)	183	3(1.6)
40–49	89	15(16.9)	128	0(0.0)
50–59	44	10(22.7)	55	0(0.0)
60+	34	2(5.9)	42	0(0.0)
**Total**	**1219**	**105(8.6)**	**1681**	**30(1.8)**
Statistic (F test)	F = 21.2;p<0.001		F = 1.0;p = 0.42	

All individuals have given blood samples for DAT testing; even though all individuals had LST administered, only 1220 individuals came back for reading skin indurations within 48–72 hours.

The age of one individual was not documented.

**Table 3:** Prevalence of LST and DAT positivity shown by districts (Hamar and Banna-Tsamai) and gender in the central region of South Omo, South West Ethiopia, July 2013.

Districts	LST		DAT	
	# tested	# (%) positive	# tested	# (%) positive
**Hamar**	690	74 (10.7)	971	25 (2.6)
• Females	384	38 (9.9)	537	7 (1.3)
• Males	306	36 (11.8)	434	18 (4.1)
**Banna-Tsamai**	530	31 (5.8)	711	5 (0.7)
• Females	286	22 (7.7)	388	2 (0.5)
• Males	244	9 (3.7)	323	3 (0.9)
**Both Districts**	1220	105 (8.6)	1682	30 (1.8)
• Females	670	60 (9.0)	925	9 (1.0)
• Males	550	45 (8.2)	757	21 (2.8)
**Pearson Chi-square** (for comparisons by District)	X^2^ = 9.06; p < 0.01		X^2^ = 8.21; p < 0.01	
**Pearson Chi-square** (for comparison by gender [M vs. F])	X^2^ = 0.23; p = 0.63		X^2^ = 7.71; p < 0.01	

T = Total; H = Hamar District; B-T = Banna-Tsamai District; M = Males; F = Females

Variations in the prevalence of LST positivity in different study villages/clusters, as shown in Table 3, depicted highest prevalence of 22.6% in Wisna; followed by Ayro (15.0%) and Luka (12.1%). When comparing the two districts, LST positive ratein Hamar was significantly higher than in Banna-Tsamai (P< 0.01, Table 3). No positive LST individuals were found in Bashaw, Seliya and Guge villages (Table 1). The difference in leishmanin positivity by sampling sites (village) was statistically significant (x^2^ = 74.5: P <0.001)

In terms of age of participants, the highest rates of LST positivity were found among adolescents and adults with rates of 21.7% in the age interval 20–29; 21.0% in the 30–39; and 22.7% in the 50–59 age groups (Table 2). The lowest positivity rate was observed within the age group of less than 10 (0.8%) followed by of the 10–19 (6.2%). The difference in LST positivity by age group was statistically significant(F = 21.2: P<0.001).

Gender-wise, the difference in LST rates between males and females was not significant (Table 3).

### Direct Agglutination Test

Variations in the prevalence of DAT positivity were noted between different study sites. The highest sero-prevalence rate was found in Cherqeqa kebele, with Ayro and Wisnia villages respectively showing rates of 4.7% and 4.3% (Table 1). Argude, a village in Besheda kebele had a high sero-prevalence rate of 3.1%. Cherqeqa, the kebele with the highest positive rate of DAT had a concurrent highest positive rate of LST (Table 1). Among all DAT positive cases, two (one male and one female) had previous treatment history. The difference in DAT positivity by sampling sites (villages) was statistically significant (X^2^ = 31.8; P < 0.001).

Age-wise, individuals in the age group 10–19 and 20–29 had highest DAT positivity rates of 2.5–2.6% (Table 2). No DAT positive cases were found in the age groups above 40. These differences of DAT positivity by age groups was not significant (F test = 1.0; P = 0.42).

Unlike LST which showed no significant differences between males and females, sero-prevalence rates varied significantly between males (2.8%) and females (1.0%) (X^2^ = 7.71, P< 0.01; Table 3).

### LST and DAT

A 2 x 2 table of the LST and DAT data shown in Table 4 depicted significant associations between the tests; showing increased odds of becoming positive by either DAT or LST when one of the tests is positive, Odds Ratio of 4.76 (95% CI: [2.03, 11.15]). Among 27 DAT positive individuals, 29.6% were LST positive cf. 8.1% of the 1193 DAT negative individuals who were also DAT positive (Table 4). Similarly for LST, the risk of becoming DAT positive is higher among LST positives than LST negatives (7.6% versus 1.7%).

**Table 4:** Prevalence of LST and DAT positivity in DAT/LST positive and negative individuals in Hamar and Banna-Tsamai districts of South Omo Region, South West Ethiopia, July 2013.

Test results	LST(# and % positive)	DAT(# and % positive)	OR [95% CI](Chi-square)
**DAT pos. or neg. vs. LST outcome**			4.76(2.03,11.15) X^2^ = 15.5
• DAT positive (n = 27)	8(29.6%)		
• DAT negative (n = 1193)	97(8.1%)		
• Total (n = 1220)	105(8.6%)		
**LST pos. or neg. vs. DAT outcome**			4.76(2.03,11.15) X^2^ = 15.5
• LST positive (n = 105)		8(7.6)	
• LST negative (n = 1115)		19(1.7)	
• Total (n = 1220)		27(2.2)	

## Discussion

In areas of anthroponotic transmission, it is known that asymptomatic infections of leishmania in man play a significant role in transmission [3, 14]. Even in areas where VL is zoonotic, it is speculated that asymptomatic individuals could contribute to transmission [3]. Thus, it is important to determine the level of exposure and infection to leishmania in VL endemic regions.

LST is a valuable tool in detecting exposure to leishmania parasites in epidemiologic surveys and its usefulness to detect asymptomatic infection has been shown by many authors [9– 11, 15]. On the other hand, the test has several limitations, including lack of adequate sensitivity and specificity, and its inability to distinguish current from past exposure due to the longevity of positive LST reactions. However, its use in marking endemicity levels remains remarkable.

In this study, LST positivity rates varied between different age groups, gender and study sites. The high rate of positive LST among those individuals above the age of 19, except in the above 60, is typical of many endemic regions of Ethiopia. This observation is probably attributable to outdoor exposure to sand fly bites, possibly associated with occupational activities specific to adolescents and adults in Hamar, Banna and Tsamai. Similarly, the low rate of skin test positivity in the under 10 year old indicates the lower exposure of children to sand fly bites. In addition, adults would have higher cumulative exposure rates (booster exposures) as a result of life time encounters with sand flies. The high proportion of LST positivity observed during adulthood and in older age groups in this study and the relatively lower rate in those less than 10 years of age is in line with data from other studies carried out in endemic localities of Lower Awash valley in Eastern Ethiopia [10, 11]. Most VL infections in Eastern Africa region are acquired outdoors either in peri-domestic surroundings or in forests. It also takes place in farming areas of northwest Ethiopia and eastern Sudan [16].

In this study, we also observed marked micro-focal variations in LST positivity; with the highest prevalence observed in Wisina followed by Ayro and Luka villages while no LST positive cases were found in Bashe, Seliya and Guge villages (Table 1). All the study sites with high LST positivity are found at altitudes less than 500m as is the case in Middle Awash valley (Eastern Ethiopia) and in the northwest part of the country. The main vectors of VL in Ethiopia, i.e., *Phlebotomus martini* and *P*. *orientalis* as well as other suspected vectors such as *P*. *alexandri* are known to co-exist in the lowlands of Hamar and Tsamai. We have earlier reported the predominance *P*. *orientalis* in Nyangatom and Dasanetch districts; and *P*. *martini* in Woito [17] and also documented *P*. *martini* abundance in Besheda/Hamar (Jemal et al., unpublished). However, the distribution of these sand fly vector species has not yet been mapped completely. Micro-ecological differences such as altitude, soil type, presence and absence of termite mounds which serve as a resting place for sand fly vectors and the type of vegetation that favor the breeding of insect vectors might also account for the observed micro-focal variation in LST positivity. In-depth epidemiological and detailed entomological studies are needed to explain the observed variations in prevalence of LST and DAT positivity.

In this study, we found no significant differences in positive LST rates between males and females. This finding is different from reports of other studies carried out in Aba-Roba (Konso) by Hailu et al. [13] and in the middle awash valley by Ali et al. [10, 11], where LST positive rate was found to be higher in male populations. Women of Hamar, Banna and Tsamai communities are highly involved in agricultural activities including cattle herding predisposing them to sand fly bites as much as their male counterparts.

The overall LST positivity observed in this study, 8.6%, is lower than those observed in Awash Valley, and Aba-Roba plains [10,11,13]. The low rate of exposure is also reflected by the low rate of DAT positivity, indeed indicating that VL is a sporadic endemics or an emerging health problem in these districts. One wonders if the disease has been spreading eastward from Nyangatom and Dasanetch or westward from the adjoining Segen and Woitu river valleys where VL is known to be endemic.

DAT is a highly specific and sensitive test to detect anti-leishmanial antibodies. The test is simple to perform making it ideal for both field and laboratory use [18]. In this study, among those found DAT positive, two of them (one male and the other female) had a previous history of treatment. It was previously shown that DAT may detect antibodies in VL patients many years after treatment [19].

Sero-prevalence rate was highest in Besheda and Cherqeqa kebeles, where the majority of recently treated cases of VL have also originated. The highest DAT positive rates were found in Ayro, followed by Wisina and Argude villages. The presence of large scale agricultural activity in Wisina and Ayro villages might have contributed to the higher rates of DAT positivity. People living in those high DAT prevalence villages have agricultural farms alongside Woito River and spend most of their time working on the farms which might increase encounters with infective sandfly bites, specifically *P*. *martini*.

Even though DAT showed considerable variation among the study sites, the overall prevalence of 1.8% in this survey is lower than previous rates reported in Omo plains (around and Nyangatom and Dasanetch) and elsewhere [13, 20–22].

## Conclusion

In this study, we were able to note that asymptomatic infection of leishmania is focally prevalent. Localities with high positive LST or DAT rates were also those reporting the majority of VL cases. The data is suggestiveof an increasing incidence and geographic spread of VL within the Lower Omo Plains. Active transmission of VL is known to take place in adjoining districts of Konso in the East and Nyangatom and Dasanetch in the west where VL affects mainly children and adolescents. The apparent spatial clustering of infections as well as active VL, particularly in Besheda and Cherqeqa kebeles may suggest importation of the disease from adjoining endemic regions. In-depth epidemiological/molecular epidemiological and entomological studies are needed to assess the risks of a greater epidemic emerging. An emerging VL in Lower Omo plains is a threat to the ongoing socio-economic development schemes in Omo Valley and to the tourism industry in the region, notably in Hamar and Banna-Tsamai districts.

## Supporting information

S1 Table Previous cases of VL from Hamar and Banna-Tsamai districts treated in Arbaminch Hospital, 2006–12. A) Previous cases of VL from Hamar District treated in Arbaminch Hospital, 2006–12 B) Previous cases of VL from Banna-Tsamai District treated in Arbaminch Hospital, 2006–12.
(DOCX)

S2 Table Distribution of study participants in the study villages showing numbers of individuals tested by LST and DAT. Kebeles (lowest administrative units) are Besheda, Cherqeqa and Gune in Hamar District; and Luka and Olu in Banna-Tsamai District. Argude, Gune, Ayro, Wisina, Ago, Bere, Bashaw, Babo, Luka, Seliya, Bahe, Guge, Sala, and Shirgo are villages/clusters used as sampling units.
(DOCX)

## References

[1] Alvar J, Yactayo S, Bern C. Leishmaniasis and poverty. Trends Parasitol. 2006; 22(12):552–7. doi: 10.1016/j.pt.2006.09.004 17023215 10.1016/j.pt.2006.09.004

[2] de Menzes JP, Saraiva EM and da Rocha-Azevedo B. The site of the bite: Leishmania interaction with macrophages, neutrophils and the extracellular matrix in the dermis. Parasit & Vectors 2016; 9:264. 10.1186/s13071-016-1540-3 PMC4857439 27146515

[3] Alvar J, Velez ID, Bern C, Herrero M, Desjeux P, Cano J, et al Leishmaniasis worldwide and global estimates of its incidence. PLoS ONE 2012; 7(5):e35671 doi: 10.1371/journal.pone.0035671 22693548 10.1371/journal.pone.0035671 PMC3365071

[4] Hailu A, Argaw D and Boelaert M. Leishmaniasis In: Neglected Tropical Diseases—Sub-Saharan Africa. 2016; Eds. Gyapong J & Boatin B. Springer International Publishing AG., Switzerland ISSN 2194-8275; doi: 10.1007/978-3-319-25471-5 pp 87–112.

[5] FMOH. Guideline for Diagnosis, Treatment and Prevention of Leishmaniasis in Ethiopia. Second ed.; Addis Ababa, Ethiopia: Ethiopian Federal Ministry of Health; 2013.

[6] Gadisa E, Tsegaw T, Abera A, Elnaiem DE, den Boer M, Aseffa A. and Jorge A. Eco-epidemiology of visceral leishmaniasis in Ethiopia. 2015; 8:381 26187584 10.1186/s13071-015-0987-y PMC4506599

[7] Cole ACE, Cosgrove PC and Robinson G. A preliminary report of kala azar in a battalion of Kings African Rifles.Trans R Soc Trop Med & Hyg 1942; 36: 25–45.

[8] Hailu A.,Berhe N., Ali A. and Gemetchu T. The use of *Leishmania major* derived leishmanin in the skin test surveys of visceral leishmaniasis in Ethiopia. 1997; 74(1): 41–45. 9145577

[9] Berhe N, Balkew M, Gebre-Michael T, Ali A and Hailu A.Visceral leishmaniasis in the middle course of the Ethiopian rift valley. I. Clinical and leishmanin skin test surveys. 1998; 36(2): 113–122. 10214453

[10] Ali A, Berhe N, Mengistu G, Gebre-Michael T. Leishmaniasis survey in the Awash Valley: The magnitude of positive leishmanin reaction and its pattern in the Middle Awash. 2002; 16 (2):157–63.

[11] Ali A, Gebre-Michael T, Mengistu G, Balcha F. A survey on leishmaniasis and the leishmanin skin test profile in Lower Awash Valley, northeast Ethiopia. 2004; 18(3):159–163

[12] Meredith SE, Kroon NC, Sondorp E, Seaman J, Goris MG, van Ingen CW, Oosting H, Schoone GJ, Terpstra WJ and Oskam L. Leish-KIT, a stable direct agglutination test based on freeze-dried antigen for serodiagnosis of visceral leishmaniasis. J Clin Microbiol 1995; 33(7):1742–45. 7665640 10.1128/jcm.33.7.1742-1745.1995 PMC228261

[13] Hailu A, Gramiccia M, Kager PA. Visceral leishmaniasis in Aba-Roba, south-western Ethiopia: prevalence and incidence of active and subclinical infections. Ann Trop Med Parasitol 2009; 103(8):659–70. doi: 10.1179/000349809X12554106963555 20030990 10.1179/000349809X12554106963555

[14] Gadisa E, Custodio E, Canavate C, Sordo L, Abebe Z, et al Usefulness of the rK39- Immunochromatographic Test, Direct Agglutination Test, and Leishmanin Skin Test for detecting asymptomatic leishmania infection in children in a new visceral leishmaniasis focus in Amhara State, Ethiopia. 2012; 86(5): 792–798. 10.4269/ajtmh.2012.11-0196 PMC3335682 22556076

[15] Gramiccia M, Bettini S, Gradoni L, Ciarmoli P, Verrilli ML, et al Leishmaniasis in Sardinia. Leishmanin reaction in the human population of a focus of low endemicity of canine leishmaniasis. 1990; 84, 371–374. 10.1016/0035-9203(90)90322-6 2260172

[16] Gebre-Michael T, Balkew M, Berhe N, Hailu A. Mekonen Y. Further studies on the phlebotomine sandflies of the kala-azar endemic lowlands of Humera-Metema (north-west Ethiopia) with observations on their natural blood meal sources. Parasit Vectors; 2010; 3(1):6 doi: 10.1186/1756-3305-3-6 20181077 10.1186/1756-3305-3-6 PMC2829606

[17] Balkew M., Hailu A., Berhe N and Gemetchu T. Leishmaniasis in the lower Omo plains, southwest Ethiopia: The sand fly fauna. 1999; 37: 31*–*40.

[18] Silva ES, Schoone GJ, Gontijo CMF, Brazil RP, Pacheco RS, Schallig HDFH. Application of direct agglutination test (DAT) and fast agglutination screening test (FAST) for sero-diagnosis of visceral leishmaniasis in endemic area of Minas Gerais, Brazil. 2005; 4:4 doi: 10.1186/1475-9292-4-4 15955248 10.1186/1475-9292-4-4 PMC1183242

[19] Hailu A.Pre-and Post-treatment antibody levels in visceral leishmaniasis. 1990; 84(5): 673–675. 10.1016/0035-9203(90)90141-z 2278067

[20] Hailu A, Berhe N, Sisay Z, Abraham I, Medhin G. Sero-epidemiological and leishmanin skin test surveys of visceral leishmaniasis in south and southwest Ethiopia. 1996; 34(1):11–23. 8674496

[21] Hailu A, Berhe N, Yeneneh H. Visceral leishmaniasis in Gambela, western Ethiopia. 1996; 34:33–42. 8674498

[22] Khalil E, Zijlstra E. Epidemiology and clinical manifestations of *Leishmaniadonovani* infection in two villages in an endemic area in Eastern Sudan. Trop Med & Intl Health 2003; 7(1): 35–44. 10.1046/j.1365-3156.2002.00832.x 11851953

