# Supplementary material for: Visceral leishmaniasis in selected communities of Hamar and Banna-Tsamai districts in Lower Omo Valley, South West Ethiopia: Sero-epidemological and Leishmanin Skin Test Surveys
Source: PLoS One. 2018 May 24;13(5):e0197430. doi: 10.1371/journal.pone.0197430 (PMC5967802; doi:10.1371/journal.pone.0197430)
Supplement: S1 Table — A) Previous cases of VL from Hamar District treated in Arbaminch Hospital, 2006–12 B) Previous cases of VL from Banna-Tsamai District treated in Arbaminch Hospital, 2006–12. (DOCX) [file pone.0197430.s001.docx]

**S1 Table A. Previous cases of VL from Hamar District treated in Arbaminch Hospital, 2006-12**

| **Kebele** | **Village** | **# of VL cases** |
| --- | --- | --- |
| Besheda | Argude, Gune, Gembella, Tubuli | 23 |
| Cherqeqa | Ayro | 4 |
|  |  |  |
| Sinbile | Sinbile | 3 |
| Turmi | 01, Malagao, Kulimba | 3 |
| DimekaZuria | Ale, Sogola | 2 |
| Keja | Gembera | 1 |
| Asile | Lito | 1 |
| Halemba | Umbula | 1 |
| Zalagata | Bita | 1 |
| Zagarma | Shafata | 1 |
| Eria | Arzebo | 1 |
| Algude | Yatele | 1 |
| Dhomo | Gabon | 1 |
| All kebeles/villages in Hamar District | | 43 |

**S1 Table B. Previous cases of VL from Banna-Tsamai District treated in Arbaminch Hospital, 2006-12**

| **Kebele** | **Village** | **# of VL cases** |
| --- | --- | --- |
| Olu | Olu | 2 |
| Luka | Turke, Babo | 2 |
|  |  |  |
| Birayle | Birayle | 1 |
| All kebeles/villages in Banna-Tsamai District | | 5 |
