# Supplementary material for: Visceral leishmaniasis in selected communities of Hamar and Banna-Tsamai districts in Lower Omo Valley, South West Ethiopia: Sero-epidemological and Leishmanin Skin Test Surveys
Source: PLoS One. 2018 May 24;13(5):e0197430. doi: 10.1371/journal.pone.0197430 (PMC5967802; doi:10.1371/journal.pone.0197430)
Supplement: S2 Table — Kebeles (lowest administrative units) are Besheda, Cherqeqa and Gune in Hamar District; and Luka and Olu in Banna-Tsamai District. Argude, Gune, Ayro, Wisina, Ago, Bere, Bashaw, Babo, Luka, Seliya, Bahe, Guge, Sala, and Shirgo are villages/clusters used as sampling units. (DOCX) [file pone.0197430.s002.docx]

**S2 Table. Distribution of study participants in the study villages showing numbers of individuals**

**tested by LST and DAT**

| **District** | **Kebeles/villages** | **# of individuals**  **who participated**  **in the study** | **# tested**  **by LST** | **# tested**  **by DAT** |
| --- | --- | --- | --- | --- |
| Hamar | Besheda  Argude  Gune | n = 386  258  128 | n = 261  195  66 | n = 386  258  128 |
|  | Cherqeqa  Ayro  Wisina | n = 376  169  207 | n = 282  127  155 | n = 376  169  207 |
|  | Sinbile  Ago  Bere  Bashaw | n = 209  98  34  77 | n = 147  64  25  58 | n = 209  98  34  77 |
| Banna-Tsamai | Luka  Babo  Luka  Seliya | n = 484  156  213  115 | n = 347  123  141  83 | n = 484  156  213  115 |
|  | Olu  Bahe  Guge  Sala  Shirgo | n = 227  66  25  70  66 | n = 183  52  22  55  54 | n = 227  66  25  70  70 |
| **Both Districts** | **All villages** | **N = 1682** | **N = 1220** | **N = 1682** |
